# Association between categorization of emotionally-charged and neutral visual scenes and parameters of event-related potentials in carriers of different COMT, HTR2A, BDNF gene genotypes

**DOI:** 10.12688/f1000research.22503.2

**Published:** 2020-08-28

**Authors:** Elena V. Vorobyeva, Pavel N. Ermakov, Evgenij F. Borokhovski, Ekaterina M. Kovsh, Alexander S. Stoletniy

**Affiliations:** 1 Department of Psychophysiology and Clinical Psychology, Academy of Psychology and Education Sciences of Southern Federal University, Rostov-on-Don, 344006, Russian Federation; 2 Department of Education, Centre for the Study of Learning and Performance of Concordia University, Montreal, H3H 2S2, Canada

**Keywords:** Categorization of emotionally-charged and neutral visual scenes, parameters of event-related potentials, COMT, HTR2A, BDNF genes polymorphisms.

## Abstract

**Background: **This study aimed to discover the association between parameters of event-related potentials (ERPs) and categorization of images of visual scenes, both emotionally-charged and neutral, in carriers of different genotypes of the *COMT, HTR2A, BDNF* genes.

**Methods: **Electroencephalogram (EEG) and ERPs were recorded at 128 leads, with two ear referents. Images of different visual scenes were presented to the study participants sequentially on a monitor screen. The participants’ task was to examine these images and indicate what emotions (negative, neutral or positive) they elicit. Comparison of event-related potentials was carried out using unpaired Student t-test in EEGLAB toolbox.

**Results:** *COMT.* A stronger reaction, as reflected in the amplitude of the ERPs, in participants with the recessive homozygous Met/Met genotype was observed on latency around 200 ms to the stimuli, assessed as positive. Carriers of dominant homozygous Val/Val genotype had higher amplitude of 200 ms peak when assessed scene images as either neutral or negative in comparison to other genotypes. Participant with the Val/Met heterozygous genotype had higher amplitude of ERP that Met/Met group on same latency when assessed stimuli as negative.

*HTR2A* . Significant increase in negativity in the parietal-occipital regions revealed in the range of 350-420 ms in participants with the recessive homozygous A/A genotype when choosing any type of assessment, compared to carriers of the heterozygous genotype A/G and the dominant homozygous G/G genotype.

*BDNF.* Participants with Val/Val genotype categorized the visual images more thoroughly, as reflected in greater activation of the parietal-occipital zones and higher amplitude on ERP peak on 190 ms (negative assessment) and 160 ms (neutral assessment) then Val/Met carriers.

**Conclusions: **The *COMT, HTR2A, BDNF* gene different genotypes are associated with the process of categorizing emotionally charged and neutral visual scenes, and this relationship is reflected in the ERP parameters.

## Introduction

Studying hereditary factors that determine specifics of the categorization of visual images are very important for opening up new opportunities for individualizing educational experiences, i.e. those that could take into account individual visual characteristics of students. Specifically, emotions that actualize these visual images can play an essential role in the process of categorizing visual scenes. As it was shown in our previous work, particular features of image categorization may vary dependent on genetic differences among carriers at the level of genes that control the functioning of brain neurotransmitter systems ( Ermakov *et al.*, 2017; Kovsh *et al.*, 2018) .

From an evolutionary standpoint, emotions arose to serve as quick adaptive responses to the environment to increase chances of survival ( Nesse, 1990). Modern psychophysiology entertains the idea that genetic changes that affect neurotransmitter pathways can also influence the manifestations of emotions ( Alfimova *et al.*, 2019).

In this work, we studied specific parameters of event-related potentials (ERPs) and categorization of images of visual scenes, both emotionally-charged and neutral, in carriers of different genotypes of *COMT, HTR2A, BDNF* genes.

The catechol-O-methyltransferase ( *COMT*) gene encodes the production of the catechol-O-methyltransferase enzyme that regulates the inactivation of catecholamines (dopamine, norepinephrine, adrenaline). The COMT enzyme is involved in the metabolic degradation of the dopamine neurotransmitter in brain regions such as the prefrontal cortex, amygdala and striatum. Research have shown that the Val158Met polymorphism of *COMT* gene is associated with the activity of the brain stem, amygdala, basal ganglia and prefrontal regions, and also is involved in the modulation of neural substrate structures responsible for processing negative and positive emotions ( Williams *et al.*, 2010), motivation ( Åberg *et al.*, 2011), recognition of negative emotions ( Gohier *et al.*, 2014), and is associated with aggressiveness ( van Dongen *et al.*, 2018). There is a connection between Val158Met polymorphism of *COMT* gene and the thickness of the cerebral cortex in associative areas of the brain (prefrontal, parietal, and cingulate), in which dopamine content is typically high ( Williams *et al.*, 2010). As a result, carriers of the Met allele of Val158Met polymorphism of COMT gene have a thicker cortical layer in these particular areas ( Miranda *et al.*, 2019).

Our work also included genotyping of study participants on the serotonin *5-HTR2A* receptor gene and the brain neurotrophic factor gene ( *BDNF*). The genotypes for these genes (as well as for the *COMT* gene) could be represented in a homozygous dominant form, homozygous recessive and heterozygous forms. These genotypes are associated with different sensitivity of neuron receptors to the presence of neurotransmitters in the synaptic cleft; in the case of the *HTR2A* gene, the level of survival of dopaminergic neurons, while in the case of the *BDNF* gene, the nutrition of serotonergic neurons ( Ermakov *et al.*, 2017).

The serotonin receptor gene *HTR2A* is located in the 13ql4 – q21 region of the thirteenth chromosome that has two introns and three exons. It is the main excitatory G-protein-coupled serotonin receptor, and may also have an inhibitory effect on the visual and orbitofrontal cortex. Serotonin receptors encoded by the *5HTR2A* gene are found in large numbers in the hippocampus and in the anterior cerebral cortex, i.e. in structures closely related to emotional processes. In the *5HTR2A* gene, a -1438A/G polymorphism located in the promoter region is possible, which causes a change in the functional activity of the receptor depending on the presence of A or G alleles. The serotonin system is actively involved in the processes of cognitive and emotional control. It was also shown that the *HTR2A* serotonin receptor gene is involved in the emotional assessment of visual stimuli ( Mills *et al.*, 2016).

*BDNF* is seen as a common neurotrophin in the brain that has an activating effect on neuronal differentiation and synaptic neuroplasticity, and affects neuron survival in adulthood ( Chen *et al.*, 2004; Pearson-Fuhrhop *et al.*, 2009). Neurotrophins are combinations of growth factors in the brain that play a key role in neuronal plasticity. The *BDNF* gene is located on the short arm of chromosome 11 and contains Val66Met single nucleotide polymorphism (SNP), in which guanine can be replaced by adenine (G196A). Such a substitution causes the amino acid valine to be replaced by methionine in codon 66, and the alleles of this gene are called Val and Met. The Met allele interferes with the intracellular traffic of *BDNF*, reducing the secretion of neurotrophic brain factor, which is associated with the transition from plasticity to stability in neural networks ( Egan *et al.*, 2003). The Met allele affects the transport of neuropeptides within the cell and reduces depolarization-dependent secretion of *BDNF* ( Alfimova *et al.*, 2009).

Our previous work confirmed the hypothesis that there is specific induced electrical activity in the brain associated with the analyses of emotiogenic images in individuals with reduced (Val/Met genotype of the brain neurotrophic factor *BDNF*) and high (Val/Val genotype of the brain neurotrophic factor *BDNF* gene) cortical plasticity, as well as in individuals with different genotypes for the *HTR2A* serotonin receptor gene ( Ermakov *et al.*, 2017).

The aim of this work is to study parameters of ERPs while categorizing images of neutral and emotionally-charged visual scenes in carriers of different genotypes of *COMT, HTR2A, BDNF* genes.

## Methods

### Ethical statement

The use of experimental subjects is in accordance with ethical guidelines as outlined in the Declaration of Helsinki. In addition, the design of the experiment, the methodological approach, the conditions of confidentiality and use of oral consent for the subjects was performed according to the Code of Ethics of Southern Federal University (SFU; Rostov-on-Don, Russia) and approved ethically by the Academic Council of the Academy of Psychology and Pedagogy of SFU, on 22 February, 2017. Before the start, each participant was informed about the goal of the research, procedure, experimental conditions, and safety of their personal data. Oral consent was obtained from each participant before continuing with the study.

### Participants

This study involved 73 participants, of both sexes, average age 23±5 years (83% - females). The study was conducted from March 24, 2017 to May 25, 2017 at the Laboratory of Psychophysiology and Experimental Psychology, Department of Psychophysiology and Clinical Psychology, Academy of Psychology and Education Sciences, Southern Federal University (Rostov-on-Don, Russia). The participants were students of Rostov universities, including the Southern Federal University (SFU). SFU students received bonus points in their academic disciplines for their participation in the study.

### Genotype analysis of COMT, BDNF and HTR2A

DNA analysis was carried out as follows: after two hours of dry hunger, buccal epithelium was collected from the inner surface of the participant’s cheeks by two sterile cotton probes. Then, probes with biological material were immersed in a transport medium and sent to the «Biologicheskie reshenija i tehnologii» (Moscow, Russia), where DNA was extracted from clinical material using the «Proba-NK» reagent kit (ООО «DNK-tehnologii», Russia). Thermocycler CFX96 «Touch» («Bio-Rad», USA) was used for real-time polymerase chain reaction (PCR).

For analysis, we used genomic DNA preparations obtained from biological samples using DNA extraction kits with the removal of PCR inhibitors and the presence of at least 100 genomic copies in 1 μl. During the genetic examination, the following DNA sections were analyzed: 

– *COMT* catechol-O-methyltransferase gene (Genebank sequence AY341246, polymorphism 23753G> A, Val158Met, rs4680 code). Possible genotypes: Val/Val, Val/Met, Met/Met. Val158Met alleles are codominant.

– *BDNF* brain neurotrophic factor gene (GenBank sequence NG_011794, polymorphism 68690G> A Val66Met, rs6265 code). Possible genotypes: Val/Val, Val/Met, Met/Met.

– *HTR2A* serotonin receptor gene (GenBank sequence, NG_013011, polymorphism 4692G> A, rs6311 (Tr2)). Possible genotypes: G/G, G/A, A/A.

Genetic analysis showed that carriers of dominant (GG), heterozygous (GA), and minor (AA) genotypes of Tr2 polymorphism of the second type of serotonin receptor gene have similar genotypes for Tr3 polymorphism (CC, TC, TT, respectively). From now on, we will use the designation of genotypes by Tr2 polymorphism (A/G), implying the presence of a carrier of a similar genotype by Tr3 polymorphism.

The genotyping resulted in the following sample composition: COMT gene, 25 participants (34,2%) were carriers of Val/Val genotype, 41 – Val/Met (56,2%) and 7 – Met/Met (9,6%) genotypes; HTR2A gene, 19 participants were carriers of A/A genotype (26%), 29 – A/G (39,8) and 25 – G/G (34,2%); BDNF gene, 46 participants were carriers of Val/Val genotype (63%), 26 – Val/Met (35,6%) and 1 – Met/Met (1,4%).

### Procedure

The stimuli database ( Stoletniy *et al.*, 2020b) of the experiment, contained 445 images, has already been used in our previous works ( Ermakov *et al.*, 2016; Vorobyeva *et al.*, 2016). 147 of them are conditionally negative, 149 are positive, 149 are neutral. Resolution of pictures is 1024х768 (16°х12° of visual angle on the 100 cm distance). The average luminance of the images wasequal to 110 of 256 greyscale (30 cd/m2). Stimuli databaseavailable as *Extended data*.

Participants were seated in good lit room at viewing distance of 100 cm in front of the monitor. A grey background (30 cd/m ^2^) was on the monitor all the time of trials. Images were presented in center of screen with duration of 500 ms. The interstimuli interval was 500–1500 ms. Stimuli pictures were presented in random order regardless of their emotional valence. Participants were asked to examine the images and indicate emotion they elicit – by pressing the corresponding key on the keyboard: “1”– negative, “2” – neutral, and “3” – positive emotion. The next image appeared on the screen only after the subject evaluated the previous one and the interval between stimuli was completed. Additionally, participants were instructed to blink only when stimulus ended.

Electroencephalogram (EEG) recording was carried out using Neurovision-136 equipment manufactured by «MKS» (Russia), with a discretization frequency of 1000 Hz. ERPs were recorded at 128 leads, using «5–10» system, and with two ear referents. Bandpass filter of 0.5-50 Hz was applied.

### Data processing and analysis

The frequencies of emotionally different responses were processed using the REdaS Package for R, version 0.9.3., in order to calculate confidence intervals for averaged response rate (α = 0.05) ( Maier, 2015). The ERPs were processed using the EEGLAB version 4.11. – an interactive Matlab/Octave toolbox for processing electrophysiological data ( Delorme & Makeig, 2004). Before averaging, the recording was filtered to eliminate epochs of ERP with artifacts. For the analysis was taken interval between 100 ms before and 500 ms after onset of stimulus. ERPs were averaged and analyzed according to type of responses – negative, neutral and positive. An intragroup comparison of ERPs was carried out using unpaired Student t-test (p <0.05). ERPs to emotionally different responses were compared separately for samples of carriers of different genotypes for the *COMT, BDNF*, and *HTR2A* genes, e.g. in *COMT* group ERP compared as Val/Val vs Val/Met vs Met/Met carriers when they chose negative, neutral or positive answer, etc. All visualization was conducted in EEGLAB.

## Results

### Hardy-Weinberg equilibrium verification results

The results of estimating the frequency distribution of alleles and genotypes of the *COMT, BDNF* and *HTR2A* genes polymorphisms corresponded to the Hardy-Weinberg distribution. The estimation of the distribution of genotype frequencies for all loci, as well as the positions of the loci, are presented below in Table 1.

**Table 1.** The results of estimating the frequency distribution of alleles and genotypes of the *COMT, BDNF* and *HTR2A* genes polymorphisms corresponded to the Hardy-Weinberg equilibrium.

Genes	SNP	Position	Minor/major allele	Genotype frequency			PHWE
				minor	heterozygous	dominant	
*COMT*	rs4680	19963748	A/G	0,096	0,562	0,342	0,246
*BDNF*	rs6265	27658369	G/A	0,014	0,356	0,630	0,446
*HTR2A*	rs6311	46897343	G/A	0,260	0,397	0,343	0,232

*PHWE – P-value for Hardy-Weinberg equilibrium*.

### COMT gene

**Figure 1.**
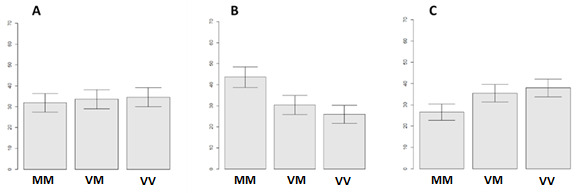
Bar graphs and confidence intervals (a = 0.05) for evaluating averaged frequencies of assessment responses to emotional stimuli for participants with different genotypes of the *COMT* gene. ( **A**) Negative stimuli, ( **B**) neutral stimuli and ( **C**) positive stimuli. MM - Met/Met, VM - Val/Met, VV - Val/Val. Left scale - average response rate in percentage.

***Behavioral results.*** The calculation of confidence intervals for assessing relative frequencies of differential responses to emotiogenic stimuli resulted in the following outcomes for participants with *COMT* genotypes. Participants with different genotypes of the *COMT* gene selected negative responses to the stimuli with approximately equal average frequency, as illustrated in Figure 1. However, the distribution of neutral and positive responses is of greater interest.

Participants with the Met/Met genotype reliably responded with the neutral reaction 10% more often than participants with Val/Met and 15% more often than Val/Val genotype carriers. Also, the same Met/Met subgroup of participants gave on average 10% and 13% fewer positive answers, respectively.

**Figure 2.**
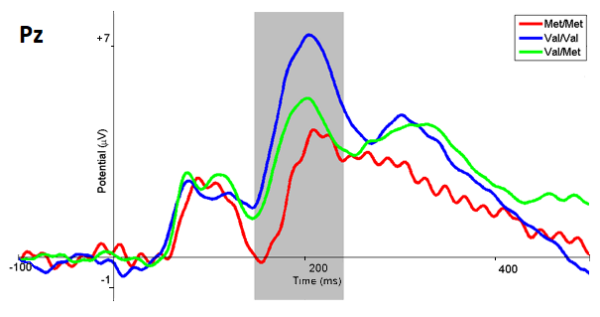
Event-related potentials (ERPs) for subgroups with different genotypes of the *COMT* gene in response to negative images. The ERP wave of the Met/Met subgroup are marked in red, Val/Val in blue, Val/Met in green, and the range with statistically significant differences in gray.

***ERP data analysis.*** The analysis of ERPs in response to images of different emotional modality showed the following. When respondents viewed the presented image as emotionally negative, the amplitude of the ERP in the parietal and occipital regions of the brain (range 150 – 250 ms) in the subgroup with the Met/Met genotype was significantly lower (p <0.03, t = -2.40) than in the Val/Met subgroup ( Figure 2). Comparison of ERP for the Met/Met and Val/Val subgroups when the respondents choose to evaluate presented images as emotionally negative showed a significantly higher amplitude (p <0.001; t = -0.40) for the Val/Val subgroup in the parietal-occipital leads, in the range of 180 – 220 ms. The amplitude of the ERPs in response to the negative images was also significantly higher (p <0.04; t = -0.13) for the Val/Val subgroup compared to the Val/Met subgroup in the same latency.

**Figure 3.**
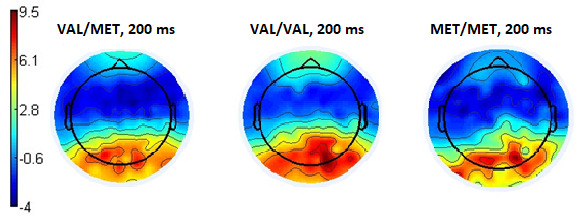
Topography of the potentials distribution at a latency of 200 ms when evaluating a stimulus as being emotionally negative, for the Val/Met, Val/Val and Met/Met subgroups of the *COMT* gene.

In addition, the topography of the potentials distribution also shows that the main differences in brain activity when evaluating a stimulus as being emotionally negative are observed mainly in the parietal-occipital regions of the cortex ( Figure 3). Examples of amplitudes values of the ERPs of the Pz electrode are presented in Table 2.

**Table 2.** Examples of amplitudes for ERPs peaks in latency and electrodes locations where statistically significant differences were found.

GENE AND GENOTYPE	TYPE OF RESPONSE / LOCATION / LATENCY/PICK AMPLITUDE		
***BDNF***	**Negative (Oz, 190 ms)**	**Neutral (Oz, 160 ms)**	**Positive (Oz, 200 ms)**
Val/Met	6,08	2,24	5,3
Val/Val	8,18	4,09	6,39
***COMT***	**Negative (Oz, 200 ms)**	**Neutral (Oz, 200 ms)**	**Positive (Oz, 200 ms)**
Met/Met	4,2	3,8	8,4
Val/Met	5,2	4,8	6,3
Val/Val	7,3	7,6	4,7
***HTR2A***	**Negative (Oz, 400 ms)**	**Neutral (Oz, 400 ms)**	**Positive (Oz, 400 ms)**
A/A	1,1	1,8	1,39
A/G	0,9	0,08	0,1
G/G	0,6	0,25	0,14

**Figure 4.**
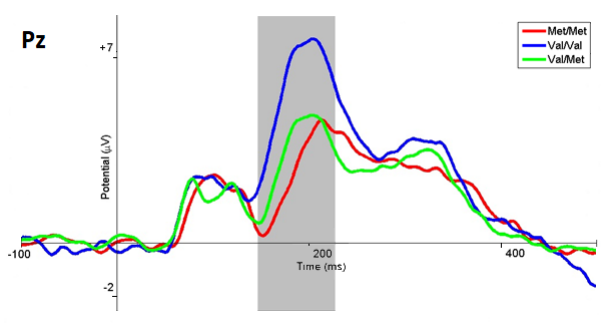
Event-related potentials (ERPs) for subgroups with different genotypes of the *COMT* gene in response to the neutral images. The ERP wave of the Met/Met subgroup are marked in red, Val/Val in blue, Val/Met in green, and the range with statistically significant differences in gray.

When the respondents evaluated emotiogenic stimuli as neutral, the following electrophysiological picture was observed. In participants with the Met/Met genotype, the amplitude of the ERP in the parietal-occipital areas within the 150 – 230 ms timeframe was significantly lower than in the Val/Met (p <0.035, t = -0.18) and Val/Val (p <0.001 t = -3.59) subgroups of participants ( Figure 4). Also, in the Val/Val genotype subgroup, the amplitude of ERPs in the same time range and region was significantly higher (p <0.07; t = -2.77) than for the Val/Met subgroup ( Figure 4). Examples of the amplitude for the Pz electrode are presented in Table 2.

**Figure 5.**
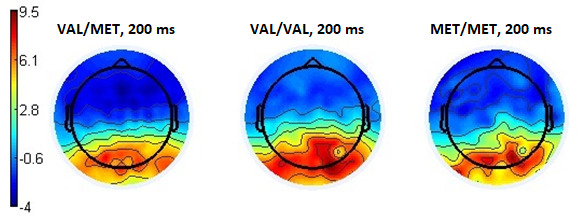
Topography of the potentials distribution at a latency of 200 ms when evaluating a stimulus as being emotionally neutral, for the Val/Met, Val/Val and Met/Met subgroups of the *COMT* gene.

The topography of the potentials distribution also shows that the main changes in brain activity associated with the selection of neutral responses appear in the parieto-occipital regions of the cortex, as is the case with the selection of negative responses ( Figure 5). Nevertheless, the amplitude and spatial differences in the latter case show that the activity of these regions in the Met/Met and Val/Met subgroups was lower, while in the Val/Val subgroup the activity was slightly higher.

**Figure 6.**
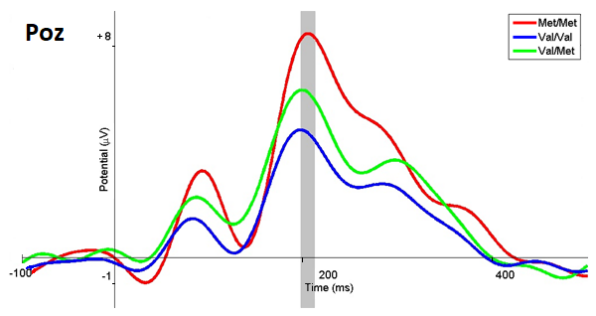
Event-related potentials (ERPs) for subgroups with different genotypes of the *COMT* gene in response to the positive images. The ERP wave of the Met/Met subgroup are marked in red, Val/Val in blue, Val/Met in green, and the range with statistically significant differences in gray.

When emotiogenic stimuli were evaluated to be positive, our analyses of the ERPs showed the following. In respondents with the Met/Met genotype of the *COMT* gene, the amplitude of ERP wave in the range from 199 to 210 ms was significantly higher than in the Val/Met subgroup (p <0.05, t = -0.09) and Val/Val (p <0.05 t = -0.28 ) subgroup ( Figure 6). In respondents with the Val/Met genotype, the ERP amplitude was significantly higher than in respondents with Val/Val (p <0.469; t = -0.74) in the same time range and region ( Figure 6). It is worth noting that in this case, the highest amplitude of the ERPs was observed in the Met/Met subgroup, and the lowest in the Val/Val subgroup, in contrast with the other two (negative and neutral) response options. Examples of the amplitude values for the POz electrode are presented in Table 2.

**Figure 7.**
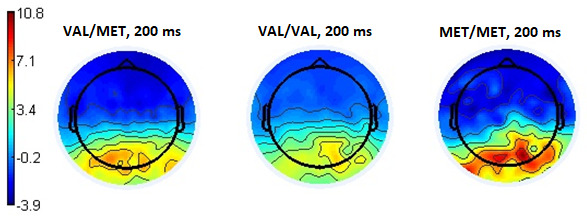
Topography of the potentials distribution at a latency of 200 ms when evaluating a stimulus as being emotionally positive, for the Val/Met, Val/Val and Met/Met subgroups of the *COMT* gene.

The topography of the distribution of cortical potentials ( Figure 7) demonstrates higher activity at a latency of 200 ms in the parieto-occipital leads for all subgroups as the trend shown in Figure 6 reflects. Nevertheless, in comparison selecting negative and neutral options, it is apparent that the choice of a positive response required less involvement of these brain areas.

Presented below are the amplitudes of ERPs at the Pz and Poz electrodes that exemplify selection of different response options when evaluating stimuli by respondents with different genotypes of the *COMT* gene. Table 2 illustrates the amplitude values of the ERPs with respect to the zero reference line.

### HTR2A gene

**Figure 8.**
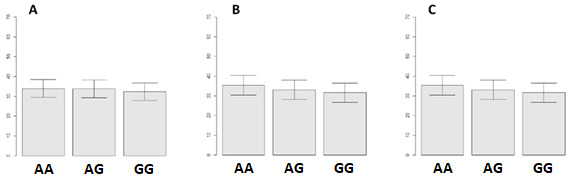
Bar graphs and confidence intervals (a = 0.05) for evaluating averaged frequencies of assessment responses to emotional stimuli for participants with different genotypes of the *HTR2A* gene. ( **A**) Negative stimuli, ( **B**) neutral stimuli and ( **C**) positive stimuli. AA – A/A, GG – G/G, AG – A/G. Left scale - average response rate in percentage.

***Behavioral results.*** The calculation of confidence intervals for the average frequency by selection of the response option showed the following for participants with *HTR2A* geneotypes. Confidence intervals overlap in all figures, which indicates the absence of any significant differences between the means ( Figure 8). This fact indicates that the participants in this sample selected responses of different emotional modality with approximately the same frequency, regardless of which genotype of *HTR2A* gene they carried.

**Figure 9.**
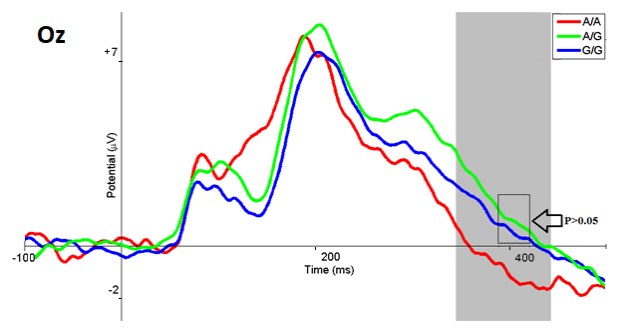
Event-related potentials (ERPs) for subgroups with different genotypes of the *HTR2A* gene in response to the negative images. The ERP wave of the A/A subgroup are marked in red, G/G in blue, A/G in green, and the range with statistically significant differences in gray.

***ERP data analysis.*** Analyses of ERPs in responses to images of different emotional modality by participants with different genotypes of the *HTR2A* gene showed the following. When a stimulus was evaluated as negatively emotionally charged, the amplitude of ERPs in the latency from 350 to 420 ms in participants with the A/A genotype showed a significantly more powerful increase in negativity than in participants with the A/G (p <0.007, t = -2.88) and G/G (p <0.022 t = -2.42) genotypes. At the same time, the ERP in subgroups of participants with the A/G and G/G genotypes, when compared with each other, did not have any significant differences (p <0.57, t = 0.56), and their amplitudes differed very slightly ( Figure 9). Examples of the amplitude values at the Oz electrode are presented in Table 2.

**Figure 10.**
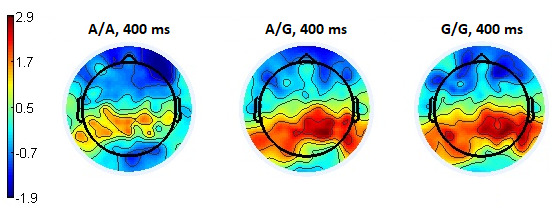
Topography of the potentials distribution at a latency of 400 ms when evaluating a stimulus as being emotionally negative, for the A/A, A/G and G/G subgroups of the *HTR2A* gene.

The topography of the ERPs for the latency of 400 ms demonstrates a high and homogeneously distributed positivity in the parieto-occipital and posterior parietal regions of the brain in subgroups with the A/G and G/G genotypes compared to the A/A subgroup ( Figure 10).

**Figure 11.**
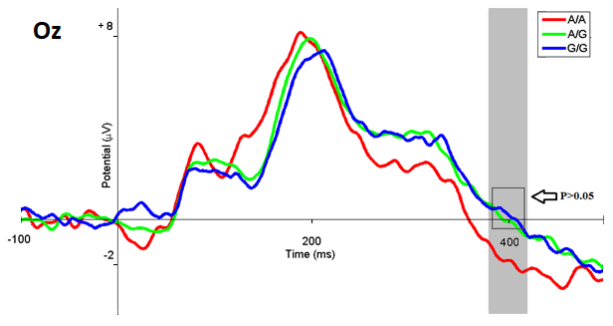
Event-related potentials (ERPs) for subgroups with different genotypes of the *HTR2A* gene in response to the neutral images. The ERP wave of the A/A subgroup are marked in red, G/G in blue, A/G in green, and the range with statistically significant differences in gray.

When a neutral response to an emotiogenic stimulus was selected in the time range from 390 to 410 ms, negativity of responses significantly increased in participants with the A/A genotype compared to the A/G (p <0.014, t = -2.58) and G/G (p < 0.008 t = -2.81) subgroups. The ERP of the A/G and G/G subgroups remained positive, and when comparing between these two subgroups, the corresponding potentials did not show significant differences (p <0.62, t = - 0.49) ( Figure 11). Examples of the amplitude values at the Oz electrode are presented in Table 2.

**Figure 12.**
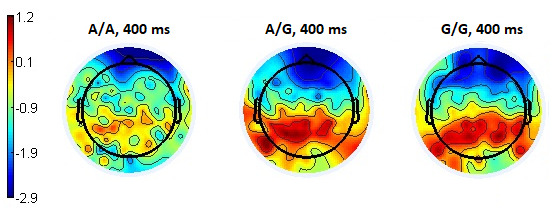
Topography of the potentials distribution at a latency of 400 ms when evaluating a stimulus as being emotionally neutral, for the A/A, A/G and G/G subgroups of the *HTR2A* gene.

The topography of the distribution of ERPs for the latency of 400 ms associated with a neutral response showed a picture similar to the situation of choosing a negative response, namely spatially uniform and higher positivity in the parietal-occipital and posterior temporal regions in subgroups with the genotypes A/G and G/G, compared with the increasing negativity in the A/A subgroup in the same brain regions ( Figure 12).

**Figure 13.**
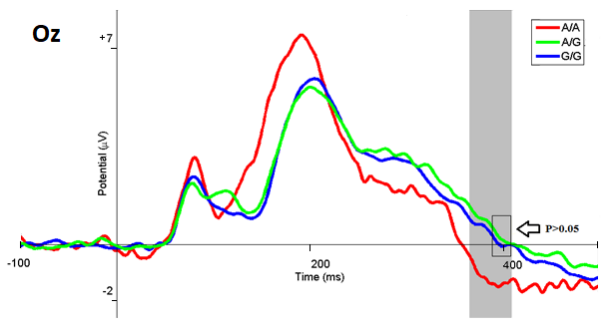
Event-related potentials (ERPs) for subgroups with different genotypes of the *HTR2A* gene in response to the positive images. The ERP wave of the A/A subgroup are marked in red, G/G in blue, A/G in green, and the range with statistically significant differences in gray.

When the visual stimuli were evaluated as emotionally positive, the amplitude of the corresponding ERPs in the latency from 370 to 405 ms in participants with the A/A genotype showed increasing negativity, significantly different from that in subgroups with the A/G (p <0.004, t = -3 , 03) and G/G (p <0.04, t = -2.11) genotypes. Comparison between the A/G and G/G subgroups revealed no significant differences (p <0.75, t = 0.31), in terms of the amplitude of the indicated latency ( Figure 13). It is useful to note ERPs of the A/G and G/G subgroups with the latency of 400 ms remained positive, but also expressed the tendency for further increase in negativity, similar to situations when participants selected responses of a different emotional valence ( Figure 9 and Figure 11). Examples of the amplitude values at the Oz electrode are presented in Table 2.

**Figure 14.**
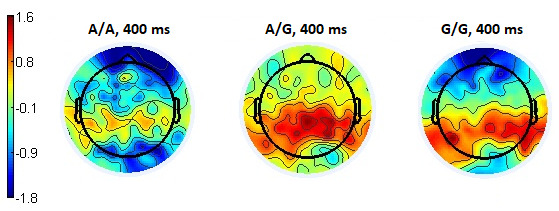
Topography of the potentials distribution at a latency of 400 ms when evaluating a stimulus as being emotionally positive, for the A/A, A/G and G/G subgroups of the *HTR2A* gene.

The topography of the distribution of ERP for the latency of 400 ms associated with positive responses showed a picture similar to the two previous situations. The parieto-occipital and posterior temporal regions showed higher positive amplitude of event-related potentials in subgroups with the A/G and G/G genotypes compared to the A/A subgroup, which had the increased negativity in these brain regions ( Figure 14).

Presented below are the amplitudes of ERPs at the Oz lead that exemplify selection of different response options when evaluating stimuli by respondents with different genotypes of the *HTR2A* gene. Table 2 illustrates the amplitude values of the ERPS with respect to the zero reference line.

### BDNF gene

**Figure 15.**
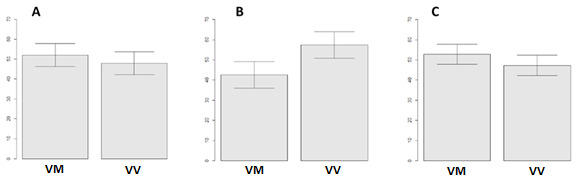
Bar graphs and confidence intervals (a = 0.05) for evaluating averaged frequencies of assessment responses to emotional stimuli for participants with different genotypes of the *BDNF* gene. ( **A**) Negative stimuli, ( **B**) neutral stimuli and ( **C**) positive stimuli. VM - Val/Met, VV - Val/Val. Left scale - average response rate in percentage.

The calculation of confidence intervals for assessing the frequency of responses different in their emotional valence was carried out only for the Val/Val and Val/Met subgroups of the *BDNF* gene as only these subgroups were sufficiently represented in the sample. Participants with the indicated genotypes on average selected negative and positive responses to the presented stimuli with an approximately equal frequency, as illustrated in Figure 15A and C. At the same time, the Val/Val subgroup chose to judge presented visual stimuli as neutral rating significantly more often (57%) than the Val/Met subgroup did ( Figure 15B).

### ERP data analysis

**Figure 16.**
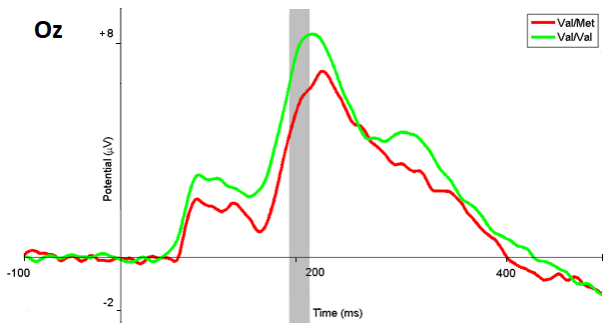
Event-related potentials (ERPs) for subgroups with different genotypes of the *BDNF* gene in response to the negative images. The ERP wave of the Val/Met subgroup are marked in red, Val/Val in green, and the range with statistically significant differences in gray.

Analyses of brain ERPs for the *BDNF* subgroup was also performed only for participants with the Val/Val and Val/Met genotypes. When the presented images were evaluated negatively emotionally charged, the amplitude of the ERPs in the parietal-occipital areas within in the 180-195 ms latency for the Val/Val subgroup was significantly higher (p <0.04, T = -2.05) than for the Val/Met subgroup ( Figure 16). Table 2 contains examples of the amplitude values for the Oz electrode with the latency of 190 ms.

**Figure 17.**
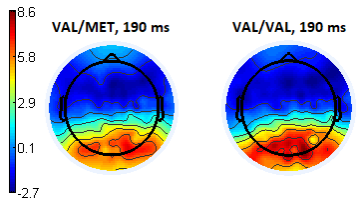
Topography of the potentials distribution at a latency of 190 ms when evaluating a stimulus as being emotionally negative, for the Val/Met, Val/Val subgroups of the *BDNF* gene.

The topography of the distribution of ERPs demonstrates a higher amplitude in the parieto-occipital regions for the Val/Val subgroup of participants. It should also be noted that in the right hemisphere, the levels of activity are slightly higher than in the left, and this is true for the participants of both subgroups ( Figure 17).

**Figure 18.**
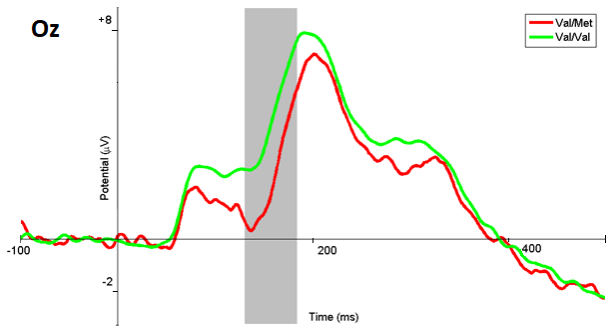
Event-related potentials (ERPs) for subgroups with different genotypes of the *BDNF* gene in response to the neutral images. The ERP wave of the Val/Met subgroup are marked in red, Val/Val in green, and the range with statistically significant differences in gray.

The analysis of the ERPs when participants evaluated visual stimuli as emotionally neutral showed significant differences in the latency of 130 – 180 ms from the stimulus onset ( Figure 18). The amplitude of the ERPs in the subgroup with the Val/Val genotype of the *BDNF* gene in this case was statistically significantly greater (p <0.005, T = –2.89) than for the subgroup with the Val/Met genotype (the amplitudes for the Oz electrode are shown in Table 2). The range of significant differences is somewhat shifted towards its earlier latency in comparison with the situations when participants were selecting a negative response.

**Figure 19.**
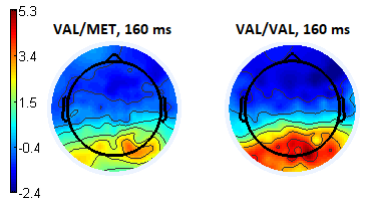
Topography of the potentials distribution at a latency of 160 ms when evaluating a stimulus as being emotionally neutral, for the Val/Met, Val/Val subgroups of the *BDNF* gene.

The topography of the distribution of cortical potentials clearly indicates their significantly higher amplitude in the parieto-occipital regions of the cortex for the subgroup with the Val/Val genotype than the subgroup with the Val/Met genotype of the *BDNF* gene at the latency of 160 ms ( Figure 19).

**Figure 20.**
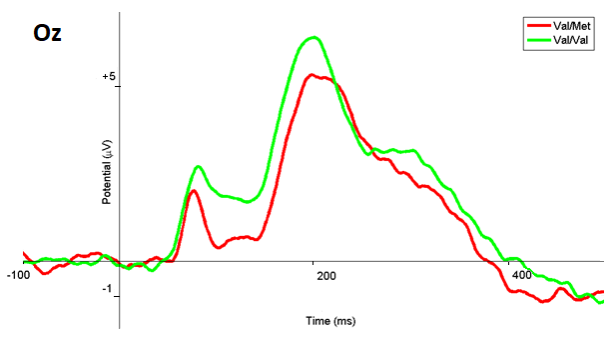
Event-related potentials (ERPs) for subgroups with different genotypes of the *BDNF* gene in response to the positive images. The ERP wave of the Val/Met subgroup are marked in red, Val/Val in green, and the range with statistically significant differences in gray.

Comparison of the ERPs in response to stimuli perceived as emotionally positive by participants with different genotypes of the *BDNF* gene did not reveal any statistically significant differences (p> 0.05, T = -1.15) ( Figure 20). Examples of the amplitude values at the Oz electrode with the latency of 200 ms are presented in Table 2.

**Figure 21.**
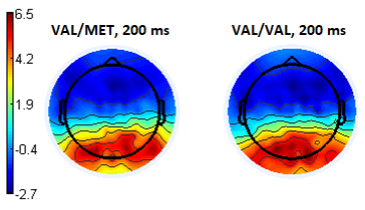
Topography of the potentials distribution at a latency of 200 ms when evaluating a stimulus as being emotionally positive, for the Val/Met, Val/Val subgroups of the *BDNF* gene.

The topography of the ERPs distribution in response to the stimuli evaluated to be emotionally positive shows an even activity of the parieto-occipital regions for 200 ms from the stimulus onset, regardless of the genotype of the *BDNF* gene ( Figure 21).

Presented below are the amplitudes of ERPs at the Oz electrode that exemplify selection of different response options when evaluating stimuli by respondents with different genotypes of the *BDNF* gene. Table 2 illustrates the amplitude values of the ERPs with respect to the zero reference line.

## Discussion

### COMT gene

A functional polymorphism of the catechol-O-methyltransferase gene ( *COMT*), Val158Met, is involved in the catecholamine system associated with the perception of emotions. Dopamine in areas, such as the prefrontal cortex, tonsil and striatum, modulates brain activity in response to aversive stimuli and regulates the processing of emotions. For example, it was found that the *COMT* Val158Met gene genotype is associated with the success of the detection of emotions, and subsequent perception of the emotions’ valence ( Tamm *et al.*, 2016).

Our work demonstrates that, when asked to assess emotional valence of visual stimuli presented to them, participants with the Met/Met genotype of the *COMT* gene more often perceived these stimuli as neutral. At the same time, participants with the Val/Val genotype of the same gene evaluated these stimuli as emotionally positive with a higher frequency. Yet, we found no statistically significant difference between the subgroups with different genotypes of the *COMT* gene in their evaluation of the presented stimuli as emotionally negative.

There is a possibility that the assessment of the stimuli as negative by the study participants may be accompanied by a fear-type reaction. It is known that individual differences in dopaminergic genotypes of the *COMT* gene can affect the initial fear reactions ( Panitz *et al.*, 2018). Another study also found lower rates in respondents with the Val/Val genotype of Val158Met *COMT* gene compared with carriers of the Met allele in fear recognition ( Heller *et al.*, 2018).

By means of the analysis of the ERPs recorded during the evaluation of the emotional valence of visually presented stimuli, it was found that there are statistically significant differences between carriers of different genotypes of the *COMT* gene for components of the ERPs in the parietal and occipital regions in the range from 150 to 250 ms. These results could be indicative of the presence of more intense processing of visual stimuli by the carriers of the Val/Val and Val/Met genotypes of the *COMT* gene in response to the stimuli perceived as neutral or negative, and by the Met/Met genotype carriers of the *COMT* gene in response to emotionally positive stimuli.

( Storozheva *et al.*, 2019), during studying auditory ERPs, found that the responses with the highest amplitude were given by people with the Val/Val genotype of the *COMT* gene, which is largely consistent with our data.

Moreover, in this work, we did not obtain statistically reliable data on the differences between the genotype subgroups of the *COMT* gene for the late components of ERPs. However, such data do exist in the periodicals. For instance, ( McLoughlin *et al.*, 2018) found that people with the Met/Met genotype of the *COMT* gene demonstrated the best task performance during the registration of event-related brain potentials, and the homozygous Met/Met genotype of the *COMT* gene was associated with a smaller frontal source of P3. The latter indicates that, although having more dopamine in the frontal areas of the cortex has advantages in some tasks, it can also jeopardize the reactive inhibition function.

Also, ( Shalev *et al.*, 2019) showed the presence of significant relationships between the *COMT* genotype and the effective threshold for visual perception, and found that insufficient or excessive catecholaminergic activity can have equally harmful effects on ability to maintain stable attention focus.

### HTR2A gene

The receptor for 5-hydroxytripamine (serotonin) 2A (5-HTR2A) is a key receptor involved in monoaminergic regulation of the basic biological functions of the body and of human behavior. A polymorphic version of the *HTR2A* rs6313 gene (102 T> C), potentially associated with the distortion of the effectiveness of post-transcriptional processes, is considered in the medical literature to be a risk factor for cognitive pathologies. A polymorphism rs6311, located in close proximity to the promoter region of the gene, is associated with changes in the expression of the *HTR2A* gene ( Zabotina *et al.*, 2018). It is assumed that anomalies in emotional regulation may result from dysfunctional serotonergic regulation of the limbic and prefrontal areas, especially of the amygdala, anterior cingulate gyrus, and the prefrontal cortex. Serotonergic genes are involved in controlling recognition of emotions ( Piel *et al.*, 2018).

The MRI data obtained in the work of ( Matsunaga *et al.*, 2017), indicates that the polymorphisms of the *HTR2A* gene are associated with individual differences in the manifestation of empathy and in experiencing happiness. Brain structures responsible for providing a mental model of empathizing with other people, include the medial prefrontal cortex, as well as the temporoparietal region. It was also found that participants with the dominant homozygous and heterozygous GG genotypes of the *HTR2A* gene experienced more pronounced feelings of happiness and greater activation of a part of the mentalization network, than people with the minor homozygous AA genotype.

( Gonzalez *et al.*, 2019) showed that the genotype of the *HTR2A* gene correlates with the manifestation of emotions of anger in a sample of adult males.

In our present work, when participants evaluated emotional valence presented to them via visual stimuli, there was no statistically significant difference in the frequency of assessing these stimuli as neutral, positive or negative in those with different genotypes of the *HTR2A* gene (A/A, A/G and G/G).

Analysis of the ERPs recorded while assessing emotional valence of presented visual stimuli found that in participants with the A/A genotype of the *HTR2A* gene, there was a statistically significant increase in negativity in the parieto-occipital regions (the time range of 350–420 ms) in comparison with participants who carried genotypes A/G and G/G of the *HTR2A* gene. This latter finding can be interpreted in support of the fact that decision-making in evaluating visual stimuli in participants with the A/A genotype of the *HTR2A* gene occurs more intensively (compared with subjects with the A/G and G/G genotypes), regardless of what response regarding emotional valence of a particular stimulus was selected by the participants.

Existing studies that feature data from the post-mortem analysis of the brain of mentally healthy people found that carriers of different alleles of the T102C polymorphic marker differ in the amount of 5HTR2A mRNA and synthesized on its basis protein product, where the carriers of the A/A genotype showed a lower level of the gene expression ( Polesskaya & Sokolov, 2002).

### BDNF gene

The interactions between genes and the environment lead to changes in the brain, depending on emotions- and behavior-transforming experiences. A positive interaction with the environment through new experiences and physical activity can engage the neuroplasticity mechanisms to improve brain functioning. Adult neurogenesis and brain neurotrophic factor (BDNF) serve as mediators of neuroplasticity ( Rogers *et al.*, 2019).

In our work, when the study participants evaluated emotional valence of visually presented stimuli, those with the Val/Val genotype of the *BDNF* gene choose the neutral response significantly more often than the participants with the Val/Met genotype did.

Our analysis of the ERPs, recorded while the participants were assessing the emotional valence of visually presented stimuli, found that when negative responses were given, the amplitude of the ERPs in the parieto-occipital regions of the Val/Val subgroup was significantly higher than that of the Val/Met subgroup in the latency of 180–195 ms. Similarly, when the participants evaluated stimuli as being emotionally neutral, the amplitude of the ERPs in the range of 130–180 ms in the parieto-occipital regions was statistically significantly higher in the subgroup with the Val/Val genotype of the *BDNF* gene than it was in the subgroup with the Val/Met genotype. No statistically significant differences between these genotypes of the *BDNF* gene were detected in response to the stimuli perceived by the participants as emotionally positive.

The obtained data can be interpreted as indicating that individuals with high cortical plasticity (i.e., with the Val/Val genotype of the *BDNF* gene), more carefully process the details of the visual stimuli, which manifests itself as an increase in activation of the parietal-occipital zones (with a statistically significantly higher peak amplitude at 190 ms of the induced activity), as it is reflected in their responses to the images estimated to be emotionally negative or neutral.

Other authors using the method of recording event-related brain potentials showed that the carriers of the Met allele of the *BDNF* gene have lower electrophysiological indicators of attention compared to the carriers of the Val allele, which is manifested in a decrease in the amplitude and increase in the latent period of the P300 component ( Getzmann *et al.*, 2013; Schofield *et al.*, 2009).

The Met allele of the Val66Met polymorphism of the *BDNF* gene is associated with the reduced levels of functioning of the amygdala and hippocampus. It also is implicated in manifestations of depression and post-traumatic stress disorder, with episodic memory deficit. There is a study that analyzed ERPs in response to recognition of emotionally negative, neutral and positive words. In it, a reduced late component P300 was observed in Met carriers compared with the carriers of the Val homozygotes in response to recognizing negatively- and positively-colored words, which could indicate that conscious experience of emotional recollection may differ depending on the *BDNF* Val66Met genotype ( Jones *et al.*, 2019).

## Conclusions

### COMT gene

As this experimental study shows, when assessing emotional valence of visually presented stimuli, participants with the recessive homozygous Met/Met genotype of the *COMT* gene at a behavioral level more often perceived presented images as emotionally neutral. In contrast, participants with the dominant Val/Val homozygous genotype of the *COMT* gene were more likely to evaluate the visual stimuli as emotionally positive. No statistically significant differences between carriers of different genotypes of the *COMT* gene were observed in response to the stimuli perceived as emotionally negative.

A stronger reaction, as reflected in the amplitude of the ERPs, in participants with the recessive homozygous Met/Met genotype of the *COMT* gene was observed to the stimuli, assessed as positive (in the parietal and occipital regions in the time range from 150 to 250 ms). In those with the dominant homozygous Val/Val genotype of the *COMT* gene, a higher amplitude of the ERPs was observed for the stimuli assessed as either neutral or negative (in the parietal and occipital regions in the time range from 150 to 250 ms). The same tendency, but to a lesser extent, was detected for the study participants with the Val/Met heterozygous genotype of the *COMT* gene.

### HTR2A gene

There were no statistically significant differences in the frequency of evaluating visually presented stimuli as either emotionally neutral, positive, or negative, as reflected in responses of the participants with different genotypes of the *HTR2A* gene (A/A, A/G, and G/G).

Analysis of the ERPs associated with participants’ evaluation of presented visual stimuli as emotionally negative, neutral, or positive revealed a statistically significant increase in negativity in the parietal-occipital regions in the range of 350–420 ms in participants with the recessive homozygous A/A genotype of the *HTR2A* gene, compared to participants with the heterozygous genotype A/G and the dominant homozygous G/G genotype of the *HTR2A* gene.

### BDNF gene

When the study participants evaluated emotional valence of visually presented stimuli, those with high cortical plasticity (carriers of the Val/Val genotype of the *BDNF* gene) chose neutral responses significantly more often than participants with reduced cortical plasticity (carriers of the Val/Met genotype).

Participants with high cortical plasticity (Val/Val genotype of the *BDNF* gene), processed the visual images more thoroughly, as reflected in greater activation of the parietal-occipital zones (with a statistically significantly higher peak amplitude at 190 ms of the ERP), which is shown to be typical for people with the homozygous Val/Val genotype when they evaluate emotional valence of visual information.

### Limitations

There are two major limitations in this study that could be addressed in future research. The first one is the overall sample size, second – insufficient amount of carriers homozygous Met/Met genotype for *BDNF* gene and partly for *COMT* gene.

The question of sample size can be interpreted in two ways. On the one hand, the specified sample size of 73 people is sufficient for a typical ERP study, since the nature of the experiment involves a significant number of trials and their averaging. On the other hand, the analysis of genetic data requires a significantly larger number of participants in order to be as accurate as possible to extrapolate to the general population. In this point of view, it seems logical to continue similar experiments on a large number of subjects for further confirmation of the obtained results.

The second limitation most likely follows from the first, since the detection of less common genotypes requires more genetic analyzes and subsequent engagement of carriers of such genotypes in the study, which is not always possible in terms of funding and time. The lack of carriers of the Met/Met genotype for the *BDNF* gene did not allow to investigate how this particular genotype influence on emotional assessment of visual scenes in contrast to other genotypes.

Both of this facts reduced the value of the article to some extent. To overcome these limitations, the next logical step in our future research is to increase the number of subjects, thereby increasing the representation of deficient genotypes.

## Data availability

### Underlying data

Open Science Framework: EEGLAB datasets for study of event-related potentials during categorization of emotionally-charged and neutral visual scenes in carriers of polymorphisms of the *COMT, HTR2A, BDNF* genes, https://doi.org/10.17605/OSF.IO/84BV6 ( Stoletniy *et al*., 2020a).

### Extended data

Open Science Framework: Stimuli database for studying emotional assessment of emotionally-charged images, https://doi.org/10.17605/OSF.IO/ZYG54 ( Stoletniy *et al*., 2020b).

Data is made available under the terms of the Creative Commons Attribution 4.0 International license (CC-BY 4.0).

## References

[ref-1] Åberg E Fandiño-Losada A Sjöholm LK: The functional Val158Met polymorphism in catechol-O-methyltransferase (COMT) is associated with depression and motivation in men from a Swedish population-based study. *J Affect Disord.* 2011;129(1–3):158–166. 10.1016/j.jad.2010.08.009 20828831

[ref-2] Alfimova MV Golimbet VE Barkhatova AN: [The role of genotype-environment interactions in the development of symptoms of anxiety and depression related to the disease burden for family]. *Zh Nevrol Psikhiatr Im S S Korsakova.* 2009;109(12):50–54. 20037522

[ref-3] Alfimova MV Golimbet VE Korovaitseva GI: The Role of the Interaction between the NMDA and Dopamine Receptor Genes in Impaired Recognition of Emotional Expression in Schizophrenia. *Neuroscience and Behavioral Physiology.* 2019;49(1):153–158. 10.1007/s11055-018-0709-y

[ref-4] Chen ZY Patel PD Sant G: Variant Brain-Derived Neurotrophic Factor (BDNF) (Met66) Alters the Intracellular Trafficking and Activity-Dependent Secretion of Wild-Type BDNF in Neurosecretory Cells and Cortical Neurons. *J Neurosci.* 2004;24(18):4401–4411. 10.1523/JNEUROSCI.0348-04.2004 15128854 PMC6729450

[ref-5] Delorme A Makeig S: EEGLAB: An open source toolbox for analysis of single-trial EEG dynamics including independent component analysis. *J Neurosci Methods.* 2004;134(1):9–21. 10.1016/j.jneumeth.2003.10.009 15102499

[ref-6] Egan MF Kojima M Callicott JH: The BDNF val66met Polymorphism Affects Activity-Dependent Secretion of BDNF and Human Memory and Hippocampal Function. *Cell.* 2003;112(2):257–269. 10.1016/s0092-8674(03)00035-7 12553913

[ref-7] Ermakov P Kovsh E Vorobyeva E: Features of induced brain activity in female carriers of various genotypes using the maoa-uVNTR marker when evaluating emotional scenes [Osobennosti vyzvannoj aktivnosti mozga devushek-nositel’nic razlichnyh genotipov po markeru MAOA -uVNTR pri ocenke jemocional’no okrashennyh scen]. *Russian Psychological Journal.* 2016;13(4):232–253. 10.21702/rpj.2016.4.14

[ref-8] Ermakov P Vorobyeva E Kovsh E: Features of evoked brain activity during recognition of emotional images in carriers of polymorphic variants of the BDNF and HTR2A genes [Osobennosti vyzvannoj aktivnosti mozga pri analize izobrazhenij jemociogennogo haraktera u nositelej polimorfnyh variantov genov BDNF i HTR2A]. *Experimental Psychology.* 2017;10(3):65–85. 10.17759/exppsy.2017100305

[ref-9] Getzmann S Gajewski PD Hengstler JG: BDNF Val66Met polymorphism and goal-directed behavior in healthy elderly - Evidence from auditory distraction. *NeuroImage.* 2013;64:290–298. 10.1016/j.neuroimage.2012.08.079 22963854

[ref-10] Gohier B Senior C Radua J: Genetic modulation of the response bias towards facial displays of anger and happiness. *Eur Psychiatry.* 2014;29(4):197–202. 10.1016/j.eurpsy.2013.03.003 23769682

[ref-11] Gonzalez I Polvillo R Ruiz‐Galdon M: Dysmorphic contribution of neurotransmitter and neuroendocrine system polymorphisms to subtherapeutic mood states. *Brain Behav.* 2019;9(2):e01140. 10.1002/brb3.1140 30656852 PMC6379594

[ref-12] Heller J Mirzazade S Romanzetti S: Impact of gender and genetics on emotion processing in Parkinson’ s disease—A multimodal study. *Neuroimage Clin.* 2018;18:305–314. 10.1016/j.nicl.2018.01.034 29876251 PMC5987844

[ref-13] Jones R Craig G Bhattacharya J: Brain-Derived Neurotrophic Factor Val66Met Polymorphism Is Associated With a Reduced ERP Component Indexing Emotional Recollection. *Front Psychol.* 2019;10:1922. 10.3389/fpsyg.2019.01922 31496979 PMC6712090

[ref-14] Kovsh EM Vorobeva EV Ermakov PN: Psychological features of Russian woman with different diplotypes of HTR2A, COMT, BDNF genes. *Behavior Genetics.* 2018;48(6):484.

[ref-16] Maier MJ: Companion package to the book R: Einführung durch angewandte Statistik R Package Version 0.9 3.2015 Reference Source

[ref-17] Matsunaga M Kawamichi H Umemura T: Neural and Genetic Correlates of the Social Sharing of Happiness. *Front Neurosci.* 2017;11:718. 10.3389/fnins.2017.00718 29311795 PMC5742108

[ref-18] McLoughlin G Palmer J Makeig S: EEG Source Imaging Indices of Cognitive Control Show Associations with Dopamine System Genes. *Brain Topogr.* 2018;31(3):392–406. 10.1007/s10548-017-0601-z 29222686 PMC5889775

[ref-19] Mills M Wieda O Stoltenberg SF: Emotion moderates the association between HTR2A (rs6313) genotype and antisaccade latency. *Exp Brain Res.* 2016;234(9):2653–2665. 10.1007/s00221-016-4669-6 27161551

[ref-20] Miranda M Morici JF Zanoni MB: Brain-Derived Neurotrophic Factor: A Key Molecule for Memory in the Healthy and the Pathological Brain. *Front Cell Neurosci.* 2019;13:363. 10.3389/fncel.2019.00363 31440144 PMC6692714

[ref-21] Nesse RM: Evolutionary explanations of emotions. *Hum Nat.* 1990;1(3):261–289. 10.1007/BF02733986 24222085

[ref-22] Panitz C Sperl MFJ Hennig J: Fearfulness, neuroticism/anxiety, and *COMT* Val158Met in long-term fear conditioning and extinction. *Neurobiol Learn Mem.* 2018;155:7–20. 10.1016/j.nlm.2018.06.001 29883709

[ref-23] Pearson-Fuhrhop KM Kleim JA Cramer SC: Brain Plasticity and Genetic Factors. *Top Stroke Rehabil.* 2009;16(4):282–299. 10.1310/tsr1604-282 19740733 PMC5800512

[ref-24] Piel JH Lett TA Wackerhagen C: The effect of 5-HTTLPR and a serotonergic multi-marker score on amygdala, prefrontal and anterior cingulate cortex reactivity and habituation in a large, healthy fMRI cohort. *Eur Neuropsychopharmacol.* 2018;28(3):415–427. 10.1016/j.euroneuro.2017.12.014 29358097

[ref-25] Polesskaya OO Sokolov BP: Differential expression of the “C” and “T” alleles of the 5-HT2A receptor gene in the temporal cortex of normal individuals and schizophrenics. *J Neurosci Res.* 2002;67(6):812–822. 10.1002/jnr.10173 11891796

[ref-26] Rogers J Renoir T Hannan AJ: Gene-environment interactions informing therapeutic approaches to cognitive and affective disorders. *Neuropharmacology.* 2019;145(Pt A):37–48. 10.1016/j.neuropharm.2017.12.038 29277490

[ref-27] Schofield PR Williams LM Paul RH: Disturbances in selective information processing associated with the BDNF Val66Met polymorphism: Evidence from cognition, the P300 and fronto-hippocampal systems. *Biol Psychol.* 2009;80(2):176–188. 10.1016/j.biopsycho.2008.09.001 18838100

[ref-28] Shalev N Vangkilde S Neville MJ: Dissociable Catecholaminergic Modulation of Visual Attention: Differential Effects of Catechol-O-Methyltransferase and Dopamine Beta-Hydroxylase Genes on Visual Attention. *Neuroscience.* 2019;412:175–189. 10.1016/j.neuroscience.2019.05.068 31195057 PMC6645579

[ref-29] Stoletniy A Kovsh E Vorobyeva E: EEGLAB datasets for study of event-related potentials during categorization of emotionally-charged and neutral visual scenes in carriers of polymorphisms of the COMT, HTR2A, BDNF genes.2020a 10.17605/OSF.IO/84BV6 PMC7495216 32983417

[ref-30] Stoletniy A Kovsh E Yavna D: Stimul database for studying emotional assessment of emotionally-charged images.2020b 10.17605/OSF.IO/ZYG54

[ref-31] Storozheva ZI Kirenskaya AV Bochkarev VK: Effects of the Val158Met Polymorphism of the Catechol-O-Methyltransferase Gene on Measures of Sensory Gating in Health and Schizophrenia. *Neuroscience and Behavioral Physiology.* 2019;49(5):595–602. 10.1007/s11055-019-00775-3

[ref-32] Tamm G Kreegipuu K Harro J: Perception of emotion in facial stimuli: The interaction of ADRA2A and COMT genotypes, and sex. *Prog Neuropsychopharmacol Biol Psychiatry.* 2016;64:87–95. 10.1016/j.pnpbp.2015.07.012 26234518

[ref-33] van Dongen JDM van Schaik RHN van Fessem M: Association between the COMT Val158Met polymorphism and aggression in psychosis: Test of a moderated mediation model in a forensic inpatient sample. *Psychology of Violence.* 2018;8(2):269–276. 10.1037/vio0000119

[ref-34] Vorobyeva E Kovsh E Yavna D: Visual evoked potentials elicited by culturally-specific images in women with different levels of hostility. *International Journal of Psychophysiology.* 2016;108:96 10.1016/j.ijpsycho.2016.07.225

[ref-35] Williams LM Gatt JM Grieve SM: COMT Val(108/158)Met polymorphism effects on emotional brain function and negativity bias . *NeuroImage.* 2010;53(3):918–925. 10.1016/j.neuroimage.2010.01.084 20139013

[ref-36] Zabotina AM Belinskaya MA Zhuravlev AS: The Influence of Rs6311 and Rs6313 Polymorphisms of Serotonin 2a Receptor Gene (HTR2A) on Its mRNA and Protein Levels in Peripheral Blood Leukocytes in Treatment with Antipsychotics. *Cell and Tissue Biology.* 2018;12(5):382–390. 10.1134/S1990519X18050115

