# Supplementary material for: Association between categorization of emotionally-charged and neutral visual scenes and parameters of event-related potentials in carriers of different COMT, HTR2A, BDNF gene genotypes
Source: F1000Res. 2020 Aug 28;9:446. Originally published 2020 May 26. [Version 2] doi: 10.12688/f1000research.22503.2 (PMC7495216; doi:10.12688/f1000research.22503.2)
Supplement: Peer review history

# Peer review: Reviewer response for version 2

**DOI:** 10.5256/f1000research.28866.r70491

**Published:** 2020-09-16

**Authors:** Sergey B. Malykh

**Affiliations:** 1 Department of Psychology, Psychological Institute of Russian Academy of Education, Lomonosov Moscow State University, Moscow, Russian Federation

I don't have any comments.

Is the work clearly and accurately presented and does it cite the current literature?

Yes

If applicable, is the statistical analysis and its interpretation appropriate?

Partly

Are all the source data underlying the results available to ensure full reproducibility?

Yes

Is the study design appropriate and is the work technically sound?

Partly

Are the conclusions drawn adequately supported by the results?

Partly

Are sufficient details of methods and analysis provided to allow replication by others?

Yes

Reviewer Expertise:

behavioral genetics

I confirm that I have read this submission and believe that I have an appropriate level of expertise to confirm that it is of an acceptable scientific standard.

# Peer review: Reviewer response for version 2

**DOI:** 10.5256/f1000research.28866.r70490

**Published:** 2020-09-01

**Authors:** Vladimir Barabanshikov

**Affiliations:** 1 Institute of Experimental Psychology, Moscow State University of Psychology and Education, Moscow, Russian Federation

The authors took into account the corrections proposed by me and made addition to the text of the article. I think the article is worthy of indexing. Good luck!

Is the work clearly and accurately presented and does it cite the current literature?

Yes

If applicable, is the statistical analysis and its interpretation appropriate?

Yes

Are all the source data underlying the results available to ensure full reproducibility?

Yes

Is the study design appropriate and is the work technically sound?

Yes

Are the conclusions drawn adequately supported by the results?

Yes

Are sufficient details of methods and analysis provided to allow replication by others?

Yes

Reviewer Expertise:

Psychophysiology, experimental psychology, general psychology

I confirm that I have read this submission and believe that I have an appropriate level of expertise to confirm that it is of an acceptable scientific standard.

# Peer review: Reviewer response for version 1

**DOI:** 10.5256/f1000research.24834.r65487

**Published:** 2020-07-06

**Authors:** Vladimir Barabanshikov

**Affiliations:** 1 Institute of Experimental Psychology, Moscow State University of Psychology and Education, Moscow, Russian Federation

The work is devoted to the actual problem of reflection of evoked potentials in the parameters of the process of evaluating visual scenes as causing positive, negative or neutral emotions in respondents. The analysis was carried out taking into account the representation of different genotypes in the study participants for the COMT, BDNF and HTR2A genes.

 Comments:

1. Describe in detail the characteristics of the study sample (average age, gender distribution).
2. It is desirable to describe the database of stimulus images (visual scenes) that was used in the work – what types of visual images were used. The work is characterized by novelty, relevance and from a scientific point of view is quite interesting. 

I recommend indexing it.

Is the work clearly and accurately presented and does it cite the current literature?

Yes

If applicable, is the statistical analysis and its interpretation appropriate?

Yes

Are all the source data underlying the results available to ensure full reproducibility?

Yes

Is the study design appropriate and is the work technically sound?

Yes

Are the conclusions drawn adequately supported by the results?

Yes

Are sufficient details of methods and analysis provided to allow replication by others?

Yes

Reviewer Expertise:

Psychophysiology, experimental psychology, general psychology

I confirm that I have read this submission and believe that I have an appropriate level of expertise to confirm that it is of an acceptable scientific standard.

# Response

**Published:** 2020-08-14

**Authors:** Alexander Stoletniy

**Affiliations:** Southern Federal University, Russian Federation

Thank you for your report!

 1.  Correction was made in the text (see "Participant")

 2.  Correction was made in the text (see "Procedure" section)

# Peer review: Reviewer response for version 1

**DOI:** 10.5256/f1000research.24834.r63932

**Published:** 2020-06-17

**Authors:** Sergey B. Malykh

**Affiliations:** 1 Department of Psychology, Psychological Institute of Russian Academy of Education, Lomonosov Moscow State University, Moscow, Russian Federation

The present article is devoted to the analysis of differences in ERP parameters depending on genetic differences (COMT, BDNF, and HTR2A gene polymorphisms) during facial recognition. The idea is interesting, however, some issues occur:

1. In the Introduction (P. 3, left and the right column) it is stated “… in carriers of polymorphisms of COMT, HTR2A, BDNF genes.” From a genetics point of view, this sentence is incorrect, since everyone has genetic polymorphism, the authors should rephrase this sentence.
2. In the Introduction (P. 3, left column) it is stated “As a result, carriers of the Met allele have a thicker cortical layer in these particular areas.” Although it is indicated later, what SNP the authors selected for the analysis, its designation (Val158Met), and functional significance have to be reported in the Introduction section. In addition, it is well documented that Val158Met alleles are codominant. Please, indicate this information also.
3. There is a mistake in the Introduction (P. 3, right column) “In the 5HTR2A gene, a 1438A/G polymorphism located in the promoter region is possible…”. Please report “-1438A/G”, since this SNP is located in the promoter gene region. Although, mention the data regarding on association of HTR2A allele with reduced/enhanced expression.
4. In Materials and Methods (P.4, left column) the authors have to transliterate or to present unique designations for the Russian phrases ≪Биологические решения и технологии≫,ООО “НекстБио”, ≪Терцик≫, ДНК-Технология, Литех, МКС.
5. In Materials and Methods it is indicated that the authors conducted real-time PCR using “Tertsik” DNA-analyzer; however, this PCR machine is not used for Real-time PCR. The authors should report the correct DNA Analyzer and the appropriate determination of genotyping. It remains unclear, why the authors did used GelDoc UV-transilluminator for TaqMan-based fluorescent PCR. They have to use Real-time DNA Analyzer.
6. In Materials and Methods Section please provide information regarding to % of women, mean age, since sex differences may modulate recognition of emotions.
7. In Materials and Methods Section (P. 4, left column) the authors use the term “mutation” for the studied SNPs instead of polymorphism or SNP.
8. In Materials and Methods Section (P. 4, left column) the authors should indicate genotype frequency of the observed genotypes.
9. Statistical analysis should be based on the appropriate statistical criterion. Since the authors deal with quantitative data (the amplitude of the ERP), the use of unpaired Student t-test requires the testing for the normality distribution and the assumption of the homogeneity of variance. Assuming small sample size (N = 73), the normality of distribution is unlikely. Please, provide required normality of distribution testing or in the case of its failure, use non-parametric statistical tests. In addition, operations with genetic data (allele and genotype frequencies) require the check for the Hardy-Weinberg disequilibrium. Please, report this information.
10. I suggest to include BDNF Met/Met-genotype together with Met/Val-genotype into one group (Met-allele carriers) for the statistical analysis.
11. The study sample is rather small (N = 73), therefore I suggest to indicate a preliminary character of conducted study and to mention this issue in Limitations.
12. The text has to be checked for the sequence of tenses.

The article can be indexed after all corrections (including appropriate statistical analysis).

Is the work clearly and accurately presented and does it cite the current literature?

Yes

If applicable, is the statistical analysis and its interpretation appropriate?

Partly

Are all the source data underlying the results available to ensure full reproducibility?

Yes

Is the study design appropriate and is the work technically sound?

Partly

Are the conclusions drawn adequately supported by the results?

Partly

Are sufficient details of methods and analysis provided to allow replication by others?

Yes

Reviewer Expertise:

behavioral genetics

I confirm that I have read this submission and believe that I have an appropriate level of expertise to confirm that it is of an acceptable scientific standard, however I have significant reservations, as outlined above.

# Response

**Published:** 2020-08-14

**Authors:** Alexander Stoletniy

**Affiliations:** Southern Federal University, Russian Federation

Thank you for your report!

**Question 1-8:** Corrections made in text of the article according to report.

**Question 9:** Hardy-Weinberg equilibrium verification results were added in the "Procedure" section. There are table 1 with frequency distribution and text with description for it. 

 Concerning about ERP statistics and normality distribution. Usually in articles with ERP method,such a question is not raised, since the technique itself, due to the number of trials (recomended more >25) and a subsuqent averaging, assumes a normal distribution of event-related potentials. For example, in our case the quantity of numeriacal data is very lagre (number of participants per number of electrodes per number of trials - 73 * 128 * 450.) Therefore, researchers are fairly free to use parametric methods without checking data for normality. This is why a T-test was used. However, a table of Shapiro Wilk normality test results for latencies at which differences were found are attached.

*Type of response*

*Shapiro-Wilk test statistics and p-value on latency (ms)*

*200             300            400*

                  Negative

 W = 0,97    W = 0,96    W = 0,98

 p = 0,16      p = 0,07     p = 0,41

                   Neutral

 W = 0,98   W = 0,97    W = 0,97

 p = 0,36     p = 0,17     p = 0,26

                  Positive

 W = 0,97   W = 0,99    W = 0,99

 p = 0,11     p = 0.95      p = 0,98

 As seen in example - all data in this latency for 73 participants and 3 responses type are normaly distributed (p>0.05)

**Question 10**: Since there was only one Met\Met genotype carriers, his\here results were excluded from research. There is a note about this in the text, in the begging of section "Results / BDNF".

**Question 11:** "Limitations" section was added in end of the article. Two majors limitations were described in the section, and futher direction of research was added.

**Question 12:** Corrections made in text of the article.
